# Ionizing radiation modulates the phenotype and function of human CD4+ induced regulatory T cells

**DOI:** 10.1186/s12865-020-00349-w

**Published:** 2020-04-16

**Authors:** Samantha S. Beauford, Anita Kumari, Charlie Garnett-Benson

**Affiliations:** Department of Biology, Georgia State University, 161 Jesse Hill Jr. Dr, Atlanta, GA 30303 USA

**Keywords:** Radiation, Regulatory T cells, T_REGS_, Cancer immunotherapy

## Abstract

**Background:**

The use of immunotherapy strategies for the treatment of advanced cancer is rapidly increasing. Most immunotherapies rely on induction of CD8+ tumor-specific cytotoxic T cells that are capable of directly killing cancer cells. Tumors, however, utilize a variety of mechanisms that can suppress anti-tumor immunity. CD4+ regulatory T cells can directly inhibit cytotoxic T cell activity and these cells can be recruited, or induced, by cancer cells allowing escape from immune attack. The use of ionizing radiation as a treatment for cancer has been shown to enhance anti-tumor immunity by several mechanisms including immunogenic tumor cell death and phenotypic modulation of tumor cells. Less is known about the impact of radiation directly on suppressive regulatory T cells. In this study we investigate the direct effect of radiation on human T_REG_ viability, phenotype, and suppressive activity.

**Results:**

Both natural and TGF-β1-induced CD4+ T_REG_ cells exhibited increased resistance to radiation (10 Gy) as compared to CD4+ conventional T cells. Treatment, however, decreased Foxp3 expression in natural and induced T_REG_ cells and the reduction was more robust in induced T_REGS_. Radiation also modulated the expression of signature iT_REG_ molecules, inducing increased expression of LAG-3 and decreased expression of CD25 and CTLA-4. Despite the disconcordant modulation of suppressive molecules, irradiated iT_REGS_ exhibited a reduced capacity to suppress the proliferation of CD8+ T cells.

**Conclusions:**

Our findings demonstrate that while human T_REG_ cells are more resistant to radiation-induced death, treatment causes downregulation of Foxp3 expression, as well as modulation in the expression of T_REG_ signature molecules associated with suppressive activity. Functionally, irradiated TGF-β1-induced T_REGS_ were less effective at inhibiting CD8+ T cell proliferation. These data suggest that doses of radiotherapy in the hypofractionated range could be utilized to effectively target and reduce T_REG_ activity, particularly when used in combination with cancer immunotherapies.

## Background

A variety of immunotherapeutic agents are being used to treat advanced malignancies and CTLA-4 and PD-1/PD-L1 T cell checkpoint blocking antibodies are currently the most common approach. Efficient tumor control by immunotherapies relies on robust CD8+ cytotoxic T lymphocyte (CTL) activity [1–3] and these immune checkpoint blocking (ICB) antibodies release the inhibitory pathways restraining the action of CTLs. While the most effective immunotherapies in development seek to generate, promote, or stimulate tumor-specific CTLs, tumors often induce an immunosuppressive microenvironment that allows them to evade immune cell killing [4]. A major mechanism of tumor-induced immunosuppression is the recruitment and/or induction of CD4+ regulatory T cells (T_REGS_) within the tumor microenvironment [5, 6].

T_REGS_ are a suppressive subset of CD4+ T cells important for preventing autoimmunity [7]. These cells are characterized by expression of the high affinity IL-2 receptor, CD25, and the transcription factor forkhead box p3 (Foxp3) [8]. T_REGS_ can be naturally derived in the thymus (nT_REG_), or they can be induced in the periphery from naïve CD4+ precursors (iT_REG_) [5, 9, 10]. Several cancer types are known to contain high levels of T_REGS_ that facilitate escape from immune surveillance [11–13]. To maintain an immunosuppressive microenvironment tumor cells have been reported to recruit peripheral T_REGS_ as well as induce conversion of CD4+ conventional T cells (T_CONV_) into T_REGS_ within the tumor [13–17]. Though nT_REG_ and iT_REG_ cells both have suppressive function, iT_REGS_ reportedly have less stable Foxp3 expression due to partial demethylation of CpG motifs within the *foxp3* locus [18]. Functionally, T_REGS_ are capable of inhibiting the proliferation and killing activity of CTLs through several mechanisms including: [a] secretion of transforming growth factor-β1(TGF-β1) and IL-10, [b] metabolic disruption through CD39 and CD73 [19], or [c] contact-dependent inhibition via cytotoxic T lymphocyte-associated antigen 4 (CTLA-4), lymphocyte activation gene 3 (LAG-3), and programmed death ligand 1 (PD-L1) signaling [20, 21].

Ionizing radiation (IR) remains a common treatment modality for most cancer types and is often used in combination with cancer immunotherapy-based strategies when radiation alone is insufficient to eradicate advanced disease [22]. Interestingly, radiation has been shown to enhance anti-tumor immune responses by several mechanisms. Research in our lab, and others, has shown that tumor cells exposed to doses within the hypofractionated range of radiation increase the expression of several cell surface proteins on tumor cells that are important for immune attack. Major histocompatibility (MHC) class I, death receptors (Fas/CD95 and TRAIL/CD253), and effector T cell costimulatory molecules (OX40L and 4-1BBL) exhibit increased expression on tumor cells surviving radiation [23–26]. Expression of these molecules subsequently promotes increased sensitivity to killing by CTLs [27, 28]. Induction of immunogenic cell death (ICD) is another mechanism of immune enhancement by radiation that results in stimulation of antigen presenting cells that can promote and drive an adaptive anti-tumor immune response [29]. In addition to local tumor control via DNA damage and cell death, radiation treatment can cause abscopal effects that result in immune control of tumors that are outside of the irradiated field [30, 31]. This phenomenon is being seen more and more frequently with the increased use of radiation in combination with immunotherapies [32, 33].

While much has been reported on the impact of IR on tumor cells, the impact of radiation on the frequency, phenotype, and suppressive function of regulatory immune cells such as T_REGS_ is less well studied. Several murine studies have shown that T_REGS_ are more radioresistant than other lymphocyte populations, however, it is less clear what effect radiotherapy (RT) has on the phenotype and function of human T_REGS_ [34, 35]. Moreover, functional studies in mice have been contradictory. Studies by Qu et al found no difference in the suppressive function of T_REGS_ from radiation treated mice compared to control mice, in contrast, Balogh et al and Billiard et al both reported decreased functional activity of irradiated T_REGS_ [36–38]. In addition, studies by Muroyama et al and Kachikwu et al reported increased T_REG_ numbers in locally irradiated tumors compared to control mice, in vivo [39, 40]. However, Cao et al (2009) and Liu et al observed decreased frequencies of human T_REGS_ irradiated in vitro and murine T_REGS_ following whole body irradiation in vivo, respectively [41, 42]. Many factors could contribute to the different outcomes reported among these studies, including differences in radiation dose used, time of evaluation after radiation, local irradiation versus whole body irradiation, and tumor-bearing versus non-tumor bearing model systems.

To more specifically extend these observations towards clinically relevant tumor immunity we sought to determine the impact of hypofractionated doses of radiation on induced human T_REGS_, as these are most likely to accumulate at tumor sites. We first assessed the direct effect of radiation on the viability and expression of Foxp3 in both nT_REG_ and iT_REG_ cells. We also evaluated the impact of radiation on the suppressive function of iT_REGS_ and the expression of molecules associated with T_REG_ functional activity: CD25, CTLA-4, LAG-3, CD39, CD73, and PD-L1. Our data reveal that radiation induces similar levels of death among human nT_REGS_ and iT_REGS_, but that less death occurs in T_REGS_ as compared to CD4+ T_CONV_ cells. We also found that radiation decreases expression of Foxp3 in both types of T_REG_ cells but that Foxp3 expression is more robustly reduced by radiation in iT_REGS_. Additionally, we show that iT_REG_ cell phenotype is directly modulated by radiation and that these cells are functionally less suppressive following radiotherapy.

## Results

### Both natural T_REG_ and induced T_REG_ cells are more resistant to cell death by radiation than CD4+ conventional T cells

**Fig. 1:**
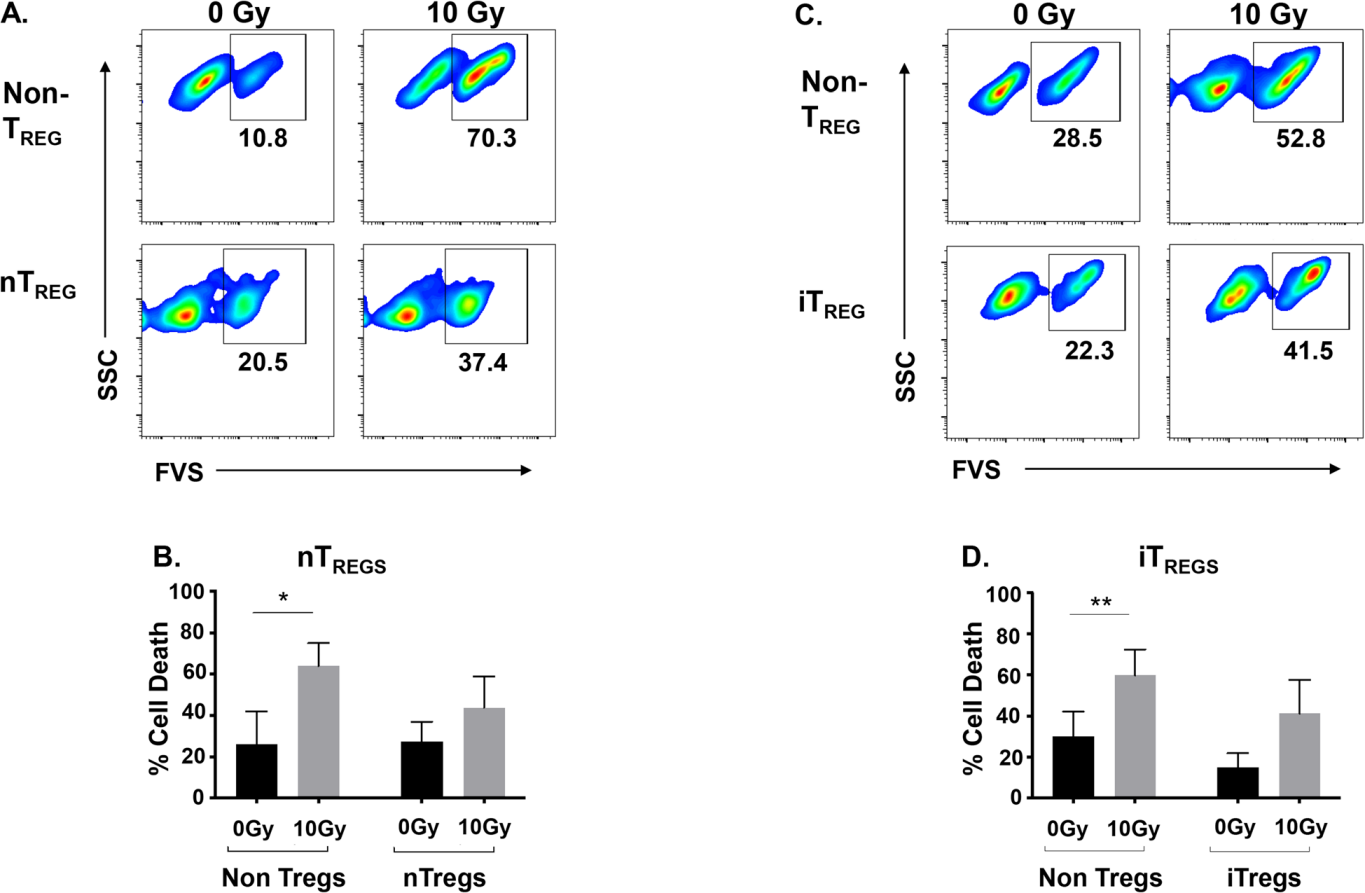
T_REGS_ are more radio-resistant than conventional CD4+ T cells. **a** Purified CD4+ T_CONV_ cells or nT_REGS_ were exposed to 10 Gy of radiation or mock irradiated (0 Gy). After 48 h, cells were stained with fixable viability stain (FVS) and CD4 or FVS, CD4, CD25, and Foxp3 to denote CD4+ T_CONV_ and T_REG_ cells, respectively. Representative plots and mean frequency (**b**) of each cell type are shown. **c** TGF-β1-induced T_REGS_ or CD4+ T_CONV_ cells were treated with 10 Gy of radiation or mock irradiated (0 Gy). 48 h post-treatment, cells were stained and analyzed by flow cytometry. Representative plots and mean frequency (**d**) of each cell type are shown. *Experiment was repeated three times with similar results. Error bars represent SEM.* *P ≤ 0.05; ** *P* ≤ 0.01; *** *P* ≤ 0.001 by paired, one-tailed Student *t* test

It has been reported that T_REG_ cells preferentially survive radiation treatment compared to CD4+ conventional T (T_CONV_) cells in mice [36, 43, 44]. In contrast, experiments utilizing human cells observed increased sensitivity of T_REGS_ to low dose radiation (< 2 Gy) [45]. Most studies exploring this question have investigated the sensitivity of natural T_REGS_ (nT_REGS_) alone or the total T_REG_ population in vivo, which potentially includes both natural and tumor induced T_REGS_. As such, the specific radio-sensitivity of induced T_REG_ (iT_REG_) cells has not been fully explored. We first compared the sensitivities of natural and induced human T_REGS_ to determine if there were differences in susceptibility to cell death between them following exposure to a hypofractionated dose of radiation. We isolated CD4 + CD25+ nT_REG_ cells from human peripheral blood mononuclear cells (PBMCs) as described in the Methods. To induce a T_REG_ phenotype, naïve CD4+ T cells were cultured in the presence of TGF-β1 and ATRA for 6 days which resulted in expression of Foxp3 and other T_REG_ associated genes [46]. nT_REG_, iT_REG_, or CD4+ T_CONV_ cells were subsequently exposed to 10 Gy of radiation and evaluated 48 h post-treatment for cell death. While CD4+ T_CONV_ cells exhibited significant increases in death after radiation, both nT_REG_ and iT_REG_ cells had lower relative amounts of cell death (Fig. 1). In separate experiments, using 7-AAD to assess viability, CD4+ T_CONV_ cells exposed to a lower dose of radiation (5 Gy) displayed around twice as much cell death as compared to T_REGS_. Irradiated CD4+ T_CONV_ cells exhibited a 4.5-fold increase in cell death over untreated cells (0 Gy) as compared to irradiated nT_REGS_ that had only a 2.3-fold increase in cell death over untreated cells. Similarly, a 1.6-fold increase in cell death over untreated cells (0 Gy) was detected in iT_REGS_ exposed to 5 Gy, as compared to a 3-fold increase in cell death observed in the control CD4+ T_CONV_ cells. These results support the idea that human T_REG_ cells are more radio-resistant as compared to CD4+ T_CONV_ cells when exposed to radiation in the hypofractionated dose range.

### Radiation decreases Foxp3 expression more robustly in iT_REGS_ as compared to nT_REGS_

**Fig. 2:**
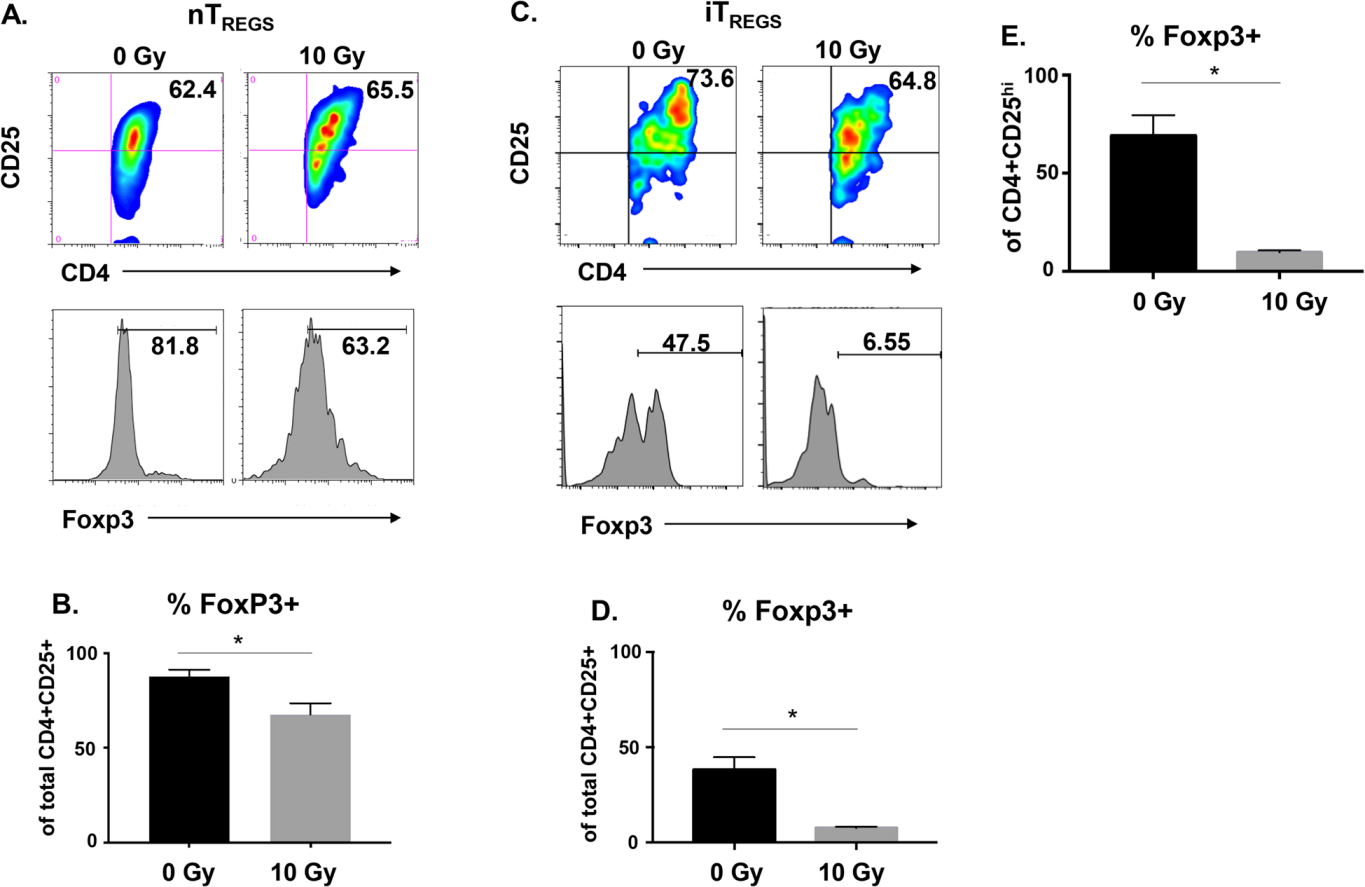
Radiation decreases Foxp3 expression in natural and induced human CD4 + CD25+ T_REGS_. nT_REG_ and iT_REG_ cells were mock irradiated or exposed to 10Gy of ionizing radiation. **a** After 48 h, nT_REGS_ were stained for expression of CD4 and CD25 by flow cytometry. **b** Expression of Foxp3 was evaluated within the CD4 + CD25+ population. **c** 48 h after irradiation, TGF-β1-induced T_REGS_ were stained for expression of CD4 and CD25 by flow cytometry. **d** Expression of Foxp3 was evaluated within the total CD4 + CD25+ population. **e** iT_REGS_ were evaluated for the expression of Foxp3 within the CD4 + CD25^hi^ population. *Experiment was repeated three times with similar results. Error bars represent SEM*. *P ≤ 0.05 by paired, one-tailed Student *t* test

T_REG_ cells express the transcription factor Foxp3, a master regulator essential for their development and suppressive function [47]. Foxp3 is the most commonly used marker for identification of T_REGS_, and while both nT_REG_ and iT_REG_ cells express it, Foxp3 expression is reportedly less stable in iT_REGS_ [18]. Therefore, it seemed plausible that nT_REGS_ and iT_REGS_ could have differential phenotypic stability following radiation treatment. Similar to studies describing the sensitivity of T_REGS_ to cell death following irradiation, most of the studies examining Foxp3 expression have been performed in mice. Murine studies have reported both an increase [39, 40] and decrease [42] in T_REG_ frequency following radiation, while data evaluating human T_REG_ cells noted a dose dependent reduction in Foxp3 expression [41]. Additionally, in vivo experiments performed in disease settings in mice evaluated the total T_REG_ population which, again, could contain both types of T_REG_ cells. In contrast, studies evaluating phenotypic changes in human cells after irradiation have been limited to nT_REG_ cells. Here, we evaluated human natural and induced T_REGS_ for Foxp3 expression following exposure to a single hypofractionated dose of radiation, in vitro. Foxp3 expression in CD4 + CD25+ nT_REGS_ decreased after treatment with 10 Gy (Fig. 2a). Foxp3 was expressed in 88% of untreated cells on average and significantly decreased to 68% in cells treated with radiation across three independent experiments (Fig. 2b). In general, more cells expressed Foxp3 in nT_REGS_ as compared to iT_REG_ (Fig. 2c), however, Foxp3 expression was further reduced in iT_REGS_ from 48% (0 Gy) to 8% (10 Gy) following radiation treatment across independent experiments (Fig. 2d). iT_REG_ cells are characterized as expressing high levels of CD25. Evaluation of the CD4 + CD25^hi^ population of iT_REGS_ revealed that Foxp3 was more highly expressed in the untreated cells of this population (69%) and that 10 Gy radiation still significantly decreased Foxp3 expression within CD25^hi^ iT_REGS_ (10%) (Fig. 2e). Interestingly, the magnitude of decreased Foxp3 expression was greater within the CD4 + CD25^hi^ population (Fig. 2e) as compared to that observed in the total CD4 + CD25+ iT_REG_ population (Fig. 2d). The percent of total CD4+ T cells remained unchanged with treatment suggesting that radiation specifically downregulates the expression of Foxp3 (data not shown). Compared to untreated cells, both nT_REGS_ and iT_REGS_ showed a significant decrease in Foxp3 expression 48 h after exposure to 10 Gy. Interestingly, iT_REGS_ showed a more robust decrease in Foxp3 expression when compared to nT_REGS_ suggesting that they are more sensitive to the effects of radiation.

### Irradiated iT_REGS_ are not converted to T_H_1 or T_H_2 cells following loss of Foxp3

**Fig. 3:**
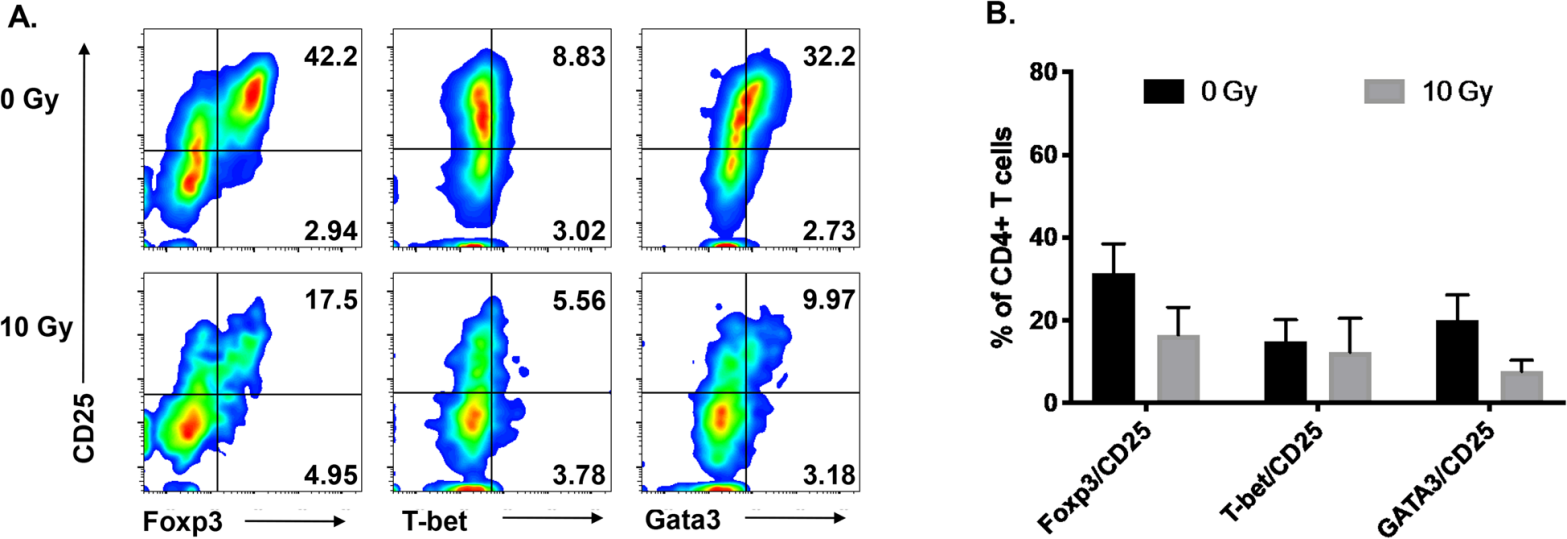
Irradiated iT_REGS_ are not converted to a T_H_1 or T_H_2 subset after radiation. Induced T_REG_ cells were mock irradiated or exposed to 10Gy of radiation. **a** iT_REGS_ were analyzed 48 h post-treatment for CD25 and Foxp3, T-bet, or GATA3 expression within CD4+ T cells. Representative plots of CD4+ T cells and (**b**) mean frequency of each subset across independent experiments. *Experiment was repeated three times with similar results. Error bars represent SEM.* *P ≤ 0.05 by paired, one-tailed Student *t* test

Plasticity is a unique characteristic of CD4+ T cells, allowing them to differentiate from one T helper (T_H_) subset to another when exposed to the right cytokine milieu [48]. Additionally, epigenetic changes in transcription factor activity induce changes in the type of CD4+ T cell needed for the appropriate immune response [49]. Foxp3 is induced in T_REG_ cells to limit cell cytotoxicity and autoimmunity [50]. The transcription factors T-box transcription factor (T-bet) and GATA binding protein 3 (GATA3) drive T_H_1 and T_H_2 differentiation, respectively [51]. Because changes in the microenvironment can directly influence the phenotype of local CD4+ T cells [52], we sought to determine if irradiated iT_REGS_ were being converted into another T_H_ subset upon downmodulation of Foxp3 expression. While radiation robustly reduced Foxp3 expression in CD4 + CD25+ iT_REG_ cells (42 to 18%), expression of T_H_1-associated T-bet or T_H_2-associated GATA3 did not exhibit a compensatory increase in expression 48 h post-treatment (Fig. 3). Interestingly, while T-bet expression was low and remained low after radiation, GATA3 expression was detected in a subpopulation of untreated cells and its expression was also reduced by radiation. These data suggest that while radiation can reduce expression of transcription factors in CD4+ T cells, irradiated Foxp3+ iT_REG_ cells are not converted into a T_H_1 or T_H_2 subset but instead can be described as an “ex-Foxp3+” CD4+ T cell.

### Radiation induces differential changes in signature T_REG_ molecules

**Fig. 4:**
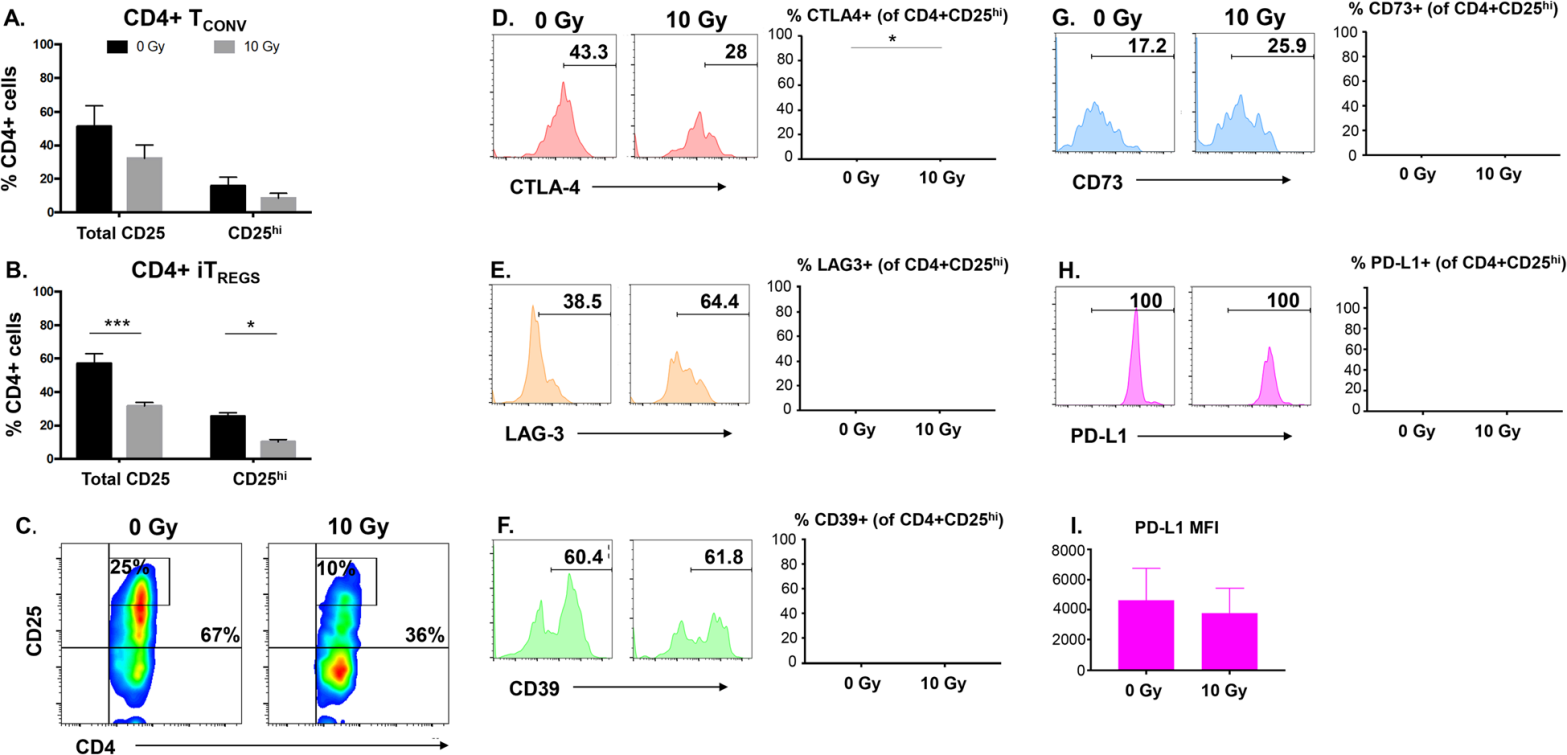
Phenotypic modulation of CD4 + CD25^hi^ iT_REGS_ by radiation. **a** CD4+ T_CONV_ cells or **b** iT_REG_ cells were mock irradiated or exposed to 10 Gy of radiation. Forty-eight hours post treatment CD4+ cells were analyzed for expression of CD25 by flow cytometry. **c** Representative plots of CD4 + CD25+ total (quadrant) or CD4 + CD25^hi^ cells (box with numbers inset in plot). The expression of (**d**) CTLA-4, (**e**) LAG-3, (**f**) CD39, (**g**) CD73, (**h**) PD-L1, and (**i**) PD-L1 mean fluorescence intensity (MFI) were evaluated within the CD4 + CD25^hi^ population 48 h after radiation*. Experiment was repeated three times with similar results. Error bars represent SEM.* *P ≤ 0.05; ** *P* ≤ 0.01; *** *P* ≤ 0.001 by paired, two-tailed Student *t* test

In addition to Foxp3, T_REG_ cells express several signature molecules associated with their regulation and functional activity. CD25, the high-affinity IL-2 receptor, is highly expressed by iT_REGS_. CD25 expression can be enhanced in T_REG_ cells by Foxp3 binding at the *Cd25* promoter [53]. Because we observed a decrease in Foxp3 following radiation treatment we wanted to determine if CD25 expression was also reduced. We first evaluated CD25 expression in CD4+ T_CONV_ cells and detected a moderate reduction in expression in irradiated cells compared to untreated cells, however, the change was not significant (Fig. 4a). In contrast, when iT_REGS_ were evaluated we observed a significant decrease in CD25 expression in irradiated cells as compared to untreated cells (Fig. 4b). This reduction in CD25 expression could be detected within the total CD4 + CD25+ population (upper right quadrant), as well as the CD4 + CD25^hi^ population (inset box gate)(Fig. 4c).

Because CD4 + CD25^hi^ iT_REG_ cells had the highest frequency of Foxp3+ cells (Fig. 2e), we further interrogated this cell population for the expression of other surface proteins associated with T_REG_ suppressive function. Cytotoxic T lymphocyte antigen 4 (CTLA-4) and lymphocyte activation gene 3 (LAG-3) have been shown to block dendritic cell maturation and inhibit effector T cell proliferation [20, 54–56]. Concordant expression of the ectoenzymes CD39 and CD73 suppress effector T cell function by converting ATP into adenosine [19]. Furthermore, the presence of PD-L1+ T_REGS_ has been correlated with exhausted effector T cells and a suppressive tumor microenvironment [21]. *CTLA-4* has been reported to be regulated by Foxp3 [57, 58]. Thus, we next sought to determine if its expression would also be reduced following radiation and found significant down-regulation of its expression from 57 to 44% across independent experiments (Fig. 4d). While LAG-3 has been reported to be regulated by Foxp3, its expression has also been detected in Foxp3-negative regulatory T cells [57, 59]. In contrast to the radiation-induced reduction seen in CD25 and CTLA4, LAG-3 expression was moderately increased across replicate experiments (34 to 48%) within the CD4 + CD25^hi^ population of cells (Fig. 4e). CD73 expression was also moderately increased though the change was not significant (20 to 28%) (Fig. 4g). CD39 was expressed in approximately half of the cells (Fig. 4f) while PD-L1 was expressed in all iT_REG_ cells (Fig. 4h). Radiation had no effect on the percent of cells expressing CD39 or PD-L1, however a small reduction in PD-L1 density was seen (Fig. 4i). These results suggest that radiation is capable of disconcordantly modulating the expression of iT_REG_-associated suppressive proteins. In addition, our findings suggest that Foxp3 regulated genes may be the most sensitive to down-regulation by radiation, and that LAG-3 is likely not regulated by Foxp3 in human iT_REGS_.

**Fig. 5:**
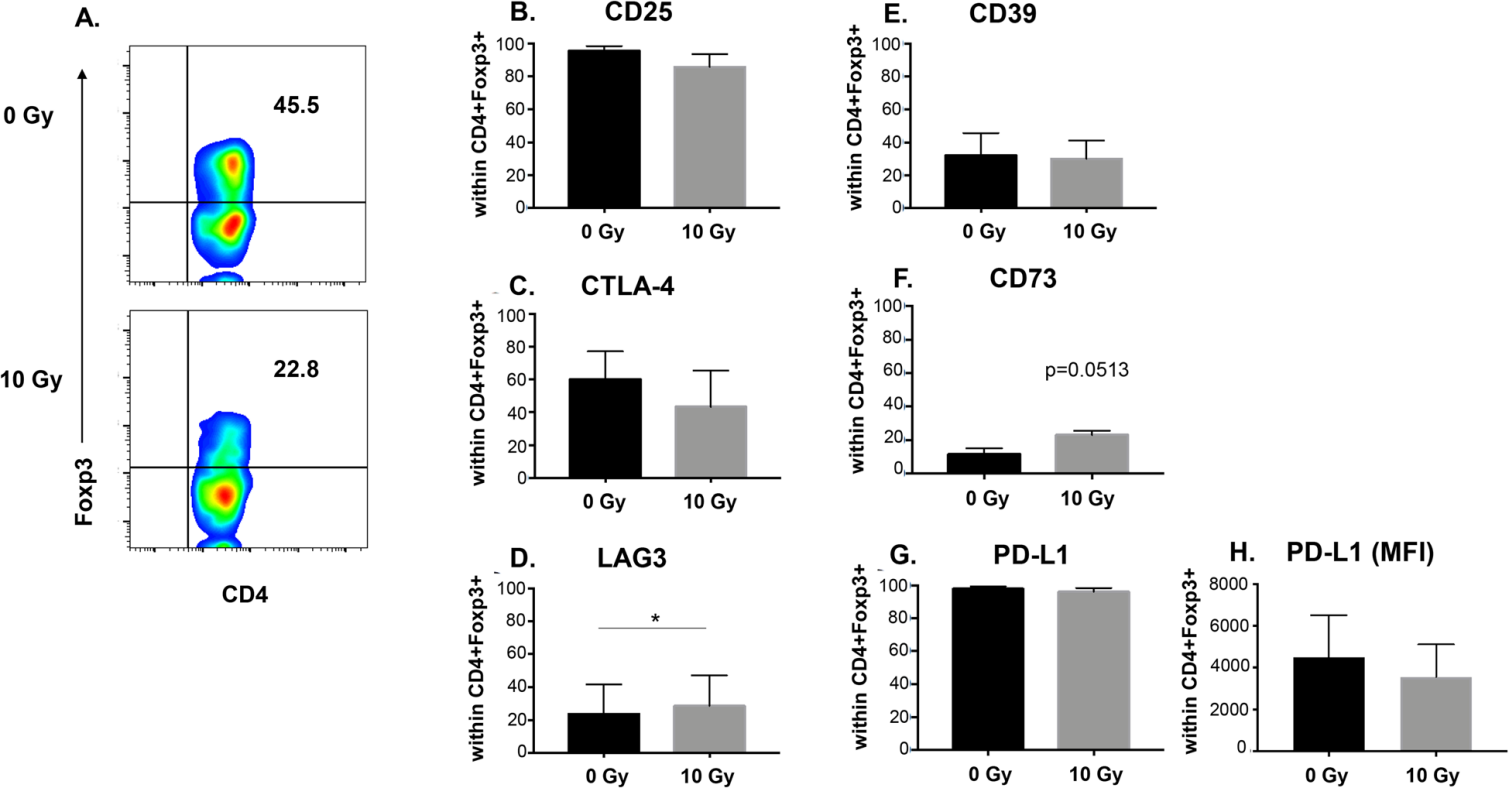
Phenotypic modulation of CD4 + Foxp3+ iT_REGS_ by radiation. **a** iT_REGS_ were mock irradiated or exposed to 10 Gy of radiation. Forty-eight hours post treatment CD4 + Foxp3+ cells were analyzed for expression of (**b**) CD25, (**c**) CTLA-4, (**d**) LAG-3, (**e**) CD39, (**f**) CD73, (**g**) PD-L1, and (**h**) PD-L1 MFI. *Experiment was repeated three times with similar results. Error bars represent SEM*. *P ≤ 0.05; ** *P* ≤ 0.01; *** *P* ≤ 0.001 by paired, two-tailed Student *t* test

Not all cells in the CD4 + CD25^hi^ population expressed Foxp3 (Fig. 2e) so we next evaluated changes in T_REG_ suppressive molecule expression within Foxp3+ cells following radiation treatment. For this analysis iT_REGS_ were defined as CD4 + Foxp3+ (Fig. 5a, upper right quadrant) and the expression of suppressive molecules after radiation was measured within this population of cells. Similar to the change detected in CD4 + CD25^hi^ cells (Fig. 4c), the expression of both CD25 (Fig. 5b) and CTLA-4 (Fig. 5c) was decreased after radiation in CD4 + Foxp3+ cells, though the modulation did not reach statistical significance. Likely because these cells were selected for expression of Foxp3, which regulates their expression. We did, however, detect a significant increase in LAG-3 expression within this cell population (Fig. 5d), as well as an increase in CD73 that neared statistical significance (Fig. 5f). Again, radiation did not alter the frequency of CD4+Foxp3+ T cells expressing either CD39 or PD-L1 (Fig. 5e and Fig. 5g) but did induce a small reduction in the density of surface PD-L1 (Fig. 5h). Overall, analysis of both CD4 + CD25^hi^ and CD4 + Foxp3+ iT_REGS_ revealed that radiation reduced expression of CTLA-4 and CD25, while conversely increasing expression of LAG-3 and CD73. However, little change in the expression of CD39 or PD-L1 was induced by in vitro irradiation in either cell population.

### Radiation inhibits suppressive activity of iT_REGS_

**Fig. 6:**
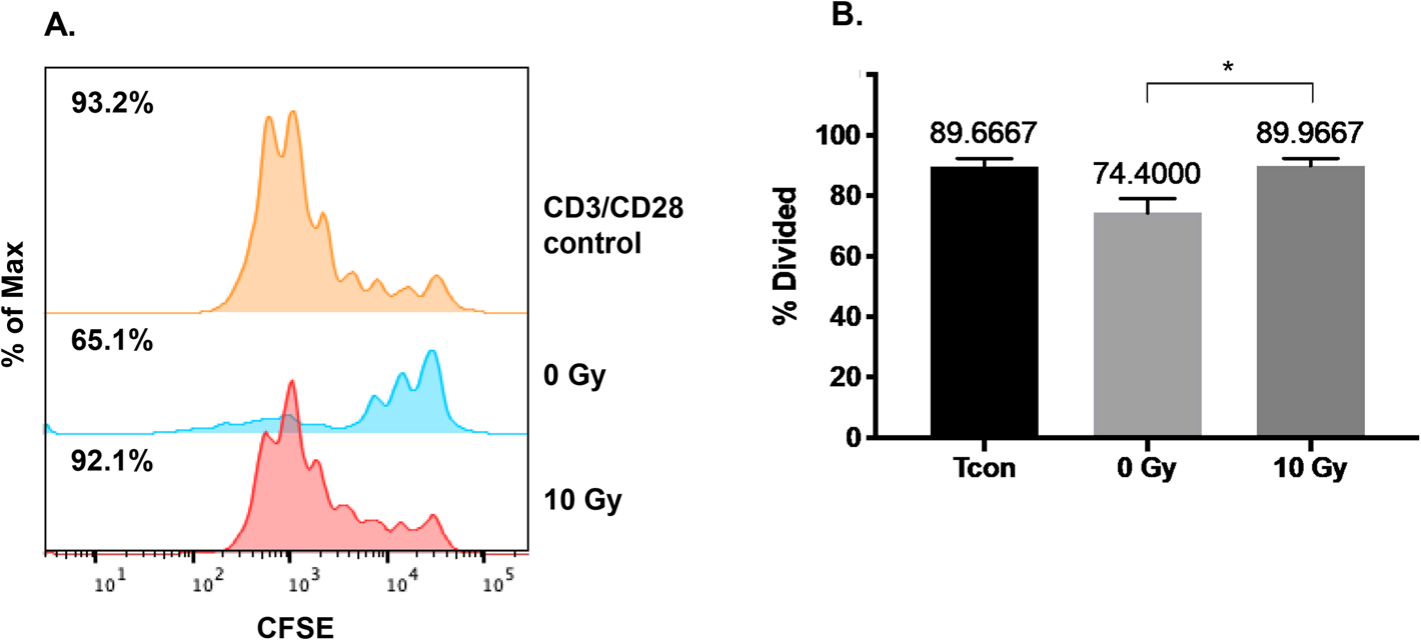
Irradiated iT_REGS_ exhibit reduced suppressive capacity. CD8+ T cells were labeled with CFSE and stimulated to proliferate with CD3 and CD28. Irradiated or non-irradiated iT_REGS_ were co-incubated with CD8+ T cells at a ratio of 1:4 for 5 days as described in Methods. **a** Histogram overlay displaying CD8+ T cell CFSE dilution alone (no added T_REGS_), in the presence of non-irradiated T_REGS_ (middle portion of plot), or irradiated iT_REGS_ (bottom portion of plot). **b** Percent of proliferating CD8+ T cells cultured alone, with mock irradiated T_REGS_ (0 Gy), or 10 Gy treated iT_REGS_. *Experiment was repeated three times with similar results. Error bars represent SEM.* *P ≤ 0.05 by one-way ANOVA with Tukey’s post-hoc analysis for multiple comparisons

We observed that radiation treatment reduced the expression of Foxp3 in iT_REG_ cells and that the expression of molecules, associated with their ability to suppress other immune cells, could be modulated both positively and negatively by IR. We next wanted to directly investigate how the suppressive function of iT_REG_ cells was affected by radiation. We compared the ability of irradiated and non-irradiated iT_REGS_ to inhibit the proliferation of CD8+ T cells. Forty-eight hours after treatment with radiation, viable iT_REGS_ were counted and co-incubated with autologous CFSE-labeled CD8+ T cells at the indicated ratio. After 5 days of co-culture, the proliferation of CD8+ T cells was measured by CFSE dilution. Ninety percent of stimulated CD8+ cells underwent cell division as indicated by reduced levels of intracellular CFSE (Fig. 6a). Proliferation of these cells was greatly reduced when iT_REGS_ were added. In addition to the percent of proliferating cells being reduced from 93 to 65%, the number of cells exhibiting more than three divisions was also reduced. In contrast, when T_REGS_ treated with 10 Gy were added the proliferation of CD8+ cells was similar to that observed in control cells (92%). Across replicate experiments, CD8+ T cells had a mean proliferation rate of 90% in the presence of 10 Gy treated iT_REGS_, as compared to only 74% following co-culture with non-irradiated iT_REG_ cells (Fig. 6b; *p* = 0.0280). Thus, iT_REG_ cells surviving 10 Gy of ionizing radiation exhibit reduced capacity to suppress the proliferation of autologous CD8+ T cells.

## Discussion

RT is a common treatment modality for cancer and is well documented to enhance antitumor immune responses by modulating tumor phenotypes, making them more susceptible to killing by CTLs [60]. The activity of CTLs, however, can be limited by suppressive T_REGS_. The effect of radiation directly on T_REG_ biology remains controversial and there are very few reports evaluating human T_REG_ cells. While T cells are known to be sensitive to high doses of radiation, the increased use of lower radiation doses per fraction, such as those used during hypofractionated radiotherapy, necessitates the need to elucidate the effects of radiation on T cells surviving RT exposure. In this study, we compared the effect of radiation treatment on human natural and induced T_REG_ cell viability and Foxp3 expression. We show that irradiated human nT_REG_ and iT_REG_ cells are more viable than irradiated CD4+ T_CONV_ cells, and that CD4 + CD25+ T_REG_ cells exhibit decreased expression of Foxp3 after exposure to ionizing radiation. We then extended our studies to further examine how the phenotype and function of iT_REG_ cells are impacted by radiation as these are likely the cells that accumulate in advanced cancers during immune escape. We demonstrate that molecules associated with T_REG_ suppressive function are differentially modulated by radiation and that the suppressive function of iT_REGS_ is inhibited.

Results here, using human cells, are in line with previous reports in mice demonstrating that T_REG_ cells are more resistant to radiation-induced cell death compared to CD4+ T_CONV_ cells [34, 44]. Additionally, we found that this resistance exists in both nT_REG_ and iT_REG_ cells (Fig. 1). Curiously, these results contrast those reported in a previous study assessing human T_REGS_ [45] exposed to much lower doses of radiation (0.94 Gy and 1.875 Gy). The authors reported significantly more cell death in T_REGS_ as compared to CD4+ T_CONV_ cells [45]. Radiation decreases human CD4+ T cell viability in a dose-dependent manner [61] and cells exposed to 5 Gy of radiation exhibit a robust decrease in live cells not detected in cells treated with ≤2 Gy. Therefore, it is plausible that human T_REGS_ are relatively more resistant to higher doses (> 2 Gy) of radiation since low dose radiation (≤ 2 Gy) did not induce significant death in CD4+ T_CONV_ cells. In the current study we selected doses, above 2 Gy, that would be relevant to those given per fraction during cancer therapy with hypofractionated RT. Moreover, most studies demonstrating the ability of RT to serve as an adjuvant for anti-tumor immunity point towards a benefit from moderate doses, around 5–12 Gy, being superior than lower 2 Gy fractions.

Much of what is known about the impact of radiation on T_REGS_ has been derived from murine models and both increased and decreased T_REG_ frequencies have been reported following radiation [39–42]. In studies evaluating T_REG_ frequency in mice, the use of whole-body versus local radiation treatment appears to have a profound effect on the number of T_REGS_ detected. Mice treated with low-dose total body irradiation (1.25 Gy) exhibited a decrease in the frequency and total number of nodal CD4 + Foxp3+ T_REG_ cells [42], while mice that received local irradiation (10 Gy and 20 Gy) were found to increase the proportion of tumoral and splenic T_REGS_ [39, 40]. It is difficult to know if the changes observed are due to the direct effect of radiation on T_REG_ cells themselves or due to changes induced in tumor cells or another immune cell type in the irradiated area. It is also unclear if the changes are due to T_REG_ cell death, redistribution to another location, or a change in the phenotypic markers used to identify the cells. Our current study, demonstrating the differential sensitivities between CD4+ T cell subsets, support the idea that in vivo observations showing increases in T_REGS_ post-RT may be detecting a decrease in the frequency of conventional CD4+ T cells which are more sensitive to RT than T_REGS_. Overall, though characterization of immune cell frequencies can provide useful information, details about the functional status of the cells, and expression of suppressive molecules, in diverse tumor model systems would be more informative. Reports of this nature have been limited. Muroyama et al recently reported that isolated tumor and splenic T_REGS_ retained their suppressive function 7 days following local irradiation in B16/F10 tumor-bearing mice [39]. Whether there was an earlier window of time during which T_REG_ function was suppressed was not explored.

Our data reveal a significant decrease in the expression of Foxp3 in human T_REG_ cells 48 h post treatment. This decrease was observed in both nT_REG_ and iT_REG_ cells but was more profound in iT_REGS_, particularly among CD25^hi^ cells (Fig. 2e). We narrowed our focus to human TGF-β1-induced T_REGS_ as these are likely to be most similar to the tumor-induced T_REGS_ that accumulate during tumor progression and immune escape. Though iT_REGS_ downregulated Foxp3, they did not alter their expression of surface CD4. In addition, the loss of Foxp3 did not appear to be due to conversion to another CD4+ T_H_ subtype as we did not detect an increase in the T_H_1 or T_H_2-associated transcription factors T-bet or GATA3 (Fig. 3). Though nT_REG_ and iT_REG_ cells both have suppressive function, iT_REGS_ have been reported to have less stable Foxp3 expression due to partial demethylation of CpG motifs within the *foxp3* locus [18]. Demethylation of the *foxp3* locus yields the gene accessible to the binding of numerous transcription factors [62]. Radiation can alter the epigenetic enzymes associated with specific gene promotors in cancer cells [63, 64] and has been reported to alter DNA methylation, both globally and in a gene-specific manner [65]. Thus, it seems reasonable that radiation could be altering the epigenetic state of the *foxp3* locus, and we would expect iT_REGS_ to be more sensitive to these changes since the region is already partially methylated. Confirmation of this mechanism warrants further investigation. Alternatively, binding of STAT5 to the T_REG_-specific demethylated region (TSDR) within the conserved noncoding sequence 2 (CNS2) has been shown to stabilize Foxp3 expression [66], and blockade of the JAK3/STAT5 signaling pathway has been demonstrated to downregulate Foxp3 expression in both human and murine T_REG_ cells [67]. It is possible that radiation alters the expression of STAT5, however, we did not observe any change in phosphorylated STAT5 following radiation treatment (unpublished data) suggesting that radiation-induced regulation of Foxp3 may be independent of STAT5. The CNS2 region, however, is bound by several other transcription factors in addition to STAT5 [62], and it is possible that radiation modulates the expression of these other factors causing the subsequent reduction in Foxp3 expression.

Beyond Foxp3, expression of CD25, CTLA-4, CD39, CD73, and LAG-3 are commonly associated with T_REG_ phenotype. Additionally, the presence of PD-L1 has been detected on both human [21] and mouse T_REGS_ [68], as well as on TGF-β1-induced T_REGS_ [69]. To our knowledge, the effect of radiation on the expression of many of these molecules in human T_REGS_ has not been characterized. T_REG_ cells are commonly defined as being Foxp3+ and CD25^hi^ and we found that CD4 + CD25^hi^ cells were the most significantly reduced following treatment with radiation (Fig. 4). This cell population also exhibited a significant decrease in CTLA-4. This observation is particularly noteworthy because it suggests that, as Foxp3 regulated genes, the reduction in CD25 and CTLA-4 expression may be directly tied to the reduction of Foxp3 expression. Moreover, there was no significant reduction in the expression of the other non-Foxp3 genes associated with T_REG_ phenotype that we evaluated (CD39, CD73, and PD-L1) (Figs. 4 and 5).

We did detect a moderate increase in CD73 and the expression of both CD39 and CD73 can be increased by TGF-β [70, 71]. Though CD73 is expressed intracellularly in humans, surface expression can be induced upon activation with high-dose IL-2 therapy [72]. However, the cells examined in our study exhibited reduced expression of the IL-2 receptor and are likely less responsive to IL-2. Thus, the moderate increase we detected in the expression of CD73 could indicate that there is increased production of TGF-β from the cells. However, the fact that we saw no increase in CD39 expression, which is also sensitive to TGF-β, suggests that another mechanism of regulation may be occurring. Hypofractionated doses of radiation have been shown to increase expression of type I interferon pathway genes associated with an inflammatory signature [73, 74]. While these observations have been made in the context of the whole tumor microenvironment, our data may reveal that radiation can directly alter the cytokines secreted from T cells, which may then modulate CD73 expression.

LAG-3 expression has also been reported to be regulated by Foxp3 [57], however, it can also be expressed in CD4 + Foxp3-negative cells indicating that it is not strictly dependent on Foxp3 for expression [59]. In our experiments, we were surprised to observe a significant increase in LAG-3 expression by radiation as opposed to the decreased expression of Foxp3, CD25, and CTLA-4 (Fig. 5d). Interestingly, chemo-radiation has been shown to increase the proportion of CD4 + LAG-3+ expressing cells in head and neck cancer patients [75] demonstrating that this effect may be clinically relevant and detectable. It is possible that radiation is directly altering expression of this gene via epigenetic mechanisms as has been reported for expression of other immune regulatory genes (OX40L and 4-BBL) in irradiated tumor cells [63]. Another possibility is that radiation is altering expression of the transcription factor early growth response gene 2 (Egr2) which has been shown to convert naïve CD4+ T cells into LAG-3-expressing T_REGS_ [76]. Notably, these LAG-3-expressing T_REGS_ were characterized as being Foxp3-negative. Our study demonstrates that radiation induces a CD4 + Foxp3-negative T cell subset from CD4 + CD25^hi^Foxp3+ iT_REGS_ (“ex-Foxp3+ cells”). While we did not detect conversion of cells towards a T_H_1 or T_H_2 subset it remains plausible that radiation treatment converts Foxp3+ iT_REGS_ to another regulatory T cell subset not evaluated here. LAG-3 expression has been reported to confer Foxp3+ regulatory T cells with greater suppressive capacity [20, 56], however, we found that irradiated iT_REG_ cells were functionally less suppressive as compared to untreated cells (Fig. 6), despite a detectable increase in LAG-3 expression. This is in line with reports showing that Egr2-transduced CD4+ T cells, which express LAG-3 and IL-10, insufficiently suppressed proliferation of responder T cells in vitro [76]. Subsequent in vivo studies, however, demonstrated that Egr2-transduced CD4+ T cells did have suppressive capacity which could suggest functional differences in the activity of LAG-3+ cells in vitro versus in vivo. This could indicate that signals, such as MHC Class II, from other immune cells are necessary to stimulate the full suppressive capacity of LAG-3+ T_REGS._

How modulation of LAG-3 expression on T cells could impact cancer immunotherapy approaches is worthy of further investigation. LAG-3 expression on CD4+ and CD8+ T_CONV_ cells is known to inhibit their expansion and effector function [77, 78]. As a result, LAG-3 blocking antibodies are currently being tested pre-clinically and clinically, and recent studies have revealed that dual treatment with anti-LAG-3 and anti-PD-1 blocking antibodies can significantly enhance the proliferation of CD4+ and CD8+ T_CONV_ cells [79]. Therefore, the combined use of radiotherapy and anti-LAG-3 blocking antibodies could greatly enhance the antitumor immune response. However, how LAG-3 signaling impacts T_REGS_ remains controversial. In a murine model of Type 1 diabetes, signaling through LAG-3 was shown to limit T_REG_ function [80] and it is unclear if antagonistic antibodies that prevent LAG-3 signaling could enhance T_REG_ suppressive function at the same time that they are promoting effector T cell activity. Further studies are needed to elucidate the effect that LAG-3 antibodies have on iT_REG_ suppressive function, particularly when used in combination with radiotherapy.

Incorporation of immune-based strategies for the treatment of cancer is becoming increasingly more common in the clinic. Current use is most often for advanced disease where the tumor microenvironment has evolved to favor survival against immune attack. This selection often involves the accumulation of suppressive T_REGS_ that help cancer cells evade immune attack by CTLs. Radiotherapy (RT) has been shown to enhance tumor attack by T cells through multiple mechanisms. RT impacts diverse cells in the microenvironment (tumor cells, immune cells, stromal cells) but the effect of the therapy on each cell type has not been fully elucidated. This is challenging to fully interrogate in vivo*,* where the impact on the independent cell types is difficult to isolate. A clearer understanding of the direct effects of radiation on suppressive subsets of immune cells can inform optimal strategies for incorporating RT to specifically serve immunotherapy strategies. In this study we found that radiation is capable of directly modulating the expression of Foxp3 and several suppressive surface molecules in human iT_REGS_. Furthermore, radiation-induced changes resulted in significantly reduced functionality of induced T_REGS_ (Fig. 6). Whether this reduced activity is simply a consequence of the reduced IL-2 signaling capacity due to lower expression of CD25, or from the lower levels of CTLA-4 expression, will require further characterization. It would also be of interest to determine how radiation impacts levels of suppressive molecules that are secreted by T_REGS_, such as TGF-β1 and IL-10, as well as how long this reduction in suppressive function is retained. Ongoing in vivo studies using radiation-treated tumor-bearing mice also demonstrate reduced T_REG_ numbers after local treatment with hypofractionated doses of RT. Even if only temporary, this reduction in T_REGS_ represents a window of opportunity during which CTL engagement with tumor cells can be manipulated. Given the conflicting observations regarding the role of LAG-3 on T_REG_ biology, future studies will need to determine the functional significance of increased LAG-3 post-RT to elucidate how LAG-3 antibodies in development can be used in combination with RT to most optimally enhance therapeutic efficacy.

## Conclusions

In summary, our study found that both human nT_REG_ and iT_REG_ cells are resistant to radiation-induced cell death and that radiation treatment reduces their expression of Foxp3. In addition, we demonstrate that radiation modulates iT_REG_ cell phenotype and inhibits their suppressive activity. These data provide a rationale for the use of radiation to specifically target Foxp3+ iT_REG_ cell function and enhance anti-tumor immune responses in combination with current immunotherapy approaches.

## Methods

### Human T cell isolation

Commercially available human peripheral blood mononuclear cells (PBMCs) were obtained from healthy donors [HemaCare and ATCC]. PBMCs were purified from buffy coats by gradient centrifugation using Lymphocyte Separation Medium [Corning]. PBMCs were rested overnight in RPMI medium containing 10% FBS and 1% Penicillin/Streptomycin prior to T cell isolation by magnetic activated cell sorting (MACS). The CD4+ T cell fraction was isolated by negative depletion from total PBMCs using the human CD4 + CD25+ Regulatory T Cell Isolation Kit [Miltenyi Biotec] according to manufacturers’ instructions. CD25+ natural T_REGS_ (nT_REGS_) were subsequently positively selected for and separated from the CD4 + CD25- naïve T cell population. Cell purity was assessed by flow cytometry staining. Cells were cultured in a 37 °C incubator with 5% CO_2_ in TexMACS medium [Miltenyi Biotec]. nT_REG_ and iT_REG_ cells were supplemented with 500 U/mL and 100 U/mL of human recombinant IL-2 [Millipore], respectively.

### iT_REG_ differentiation

iT_REG_ differentiation was performed as previously described [46]. Briefly, following MACS isolation, naïve T cells were rested for 2–8 h before plating under iT_REG_ differentiation conditions at 1.1 to 1.5 × 10^5^ cells/well in a U-bottom 96-well plate. Cells were stimulated with 5 μg/mL plate-bound anti-CD3 antibody [OKT3, NA/LE], 1 μg/mL soluble anti-CD28 antibody [CD28.2, NA/LE; BD Biosciences], and 100 U/mL IL-2. Cells stimulated with only these reagents served as “mock” control cells. For iT_REG_ differentiation, 5 ng/mL TGF-β1 [R&D Systems] and 10 nM all-trans retinoic acid [Sigma-Aldrich] were additionally added. On day 3, 100 μL of medium was removed and 100 μL of fresh medium plus growth supplements was added. Cells were then incubated for an additional 3 days.

### Irradiation

A RS-2000 biological X-ray irradiator [Rad Source Technology] was used to irradiate cells. Irradiation was performed at a dose of 2 Gy/min at voltage 160 kV and 25 mA current. On day 6, cells were washed and resuspended in fresh TexMACS medium without cytokines. Cells were kept on ice and irradiated (10 Gy) or mock-irradiated (0 Gy). Immediately following irradiation, the culture medium was replaced with fresh medium plus growth supplements minus anti-CD3 and anti-CD28.

### Flow cytometry

Anti-human antibodies were used to characterize T_REG_ cells following isolation: Foxp3-Pacific Blue, Foxp3-PE [PCH101], Gata3-PE [TWAJ] and T-bet-PE [4B10; Invitrogen]; CD4-FITC, LAG-3-PE, CD39-APC and CD73-APC [BD Biosciences]; CD4-APC, CD25-APC, CD25-PE, CTLA-4-APC and PD-L1-APC [BioLegend]. 7-aminoactinomycin D (7-AAD) [BioLegend] or Fixable Viability Stain 780 or 450 [BD Biosciences] were used to exclude dead cells according to manufacturers’ instructions. Appropriate isotype control antibodies were used, and gating was based on < 5% isotype staining. Intracellular staining was performed using the Foxp3 Transcription Factor Staining Buffer Set [Invitrogen] according to manufacturers’ instructions. Data was acquired on a BD Fortessa [Beckman Coulter] and data was analyzed using FlowJo software [TreeStar].

### In vitro proliferation assay

Responder T cell proliferation assay was performed as previously described with minor modifications [81]. Briefly, purified CD8+ T cells were labeled with 2.5 μM carboxyfluorescein succinimidyl ester (CFSE) [BD Biosciences]. Labeled CD8s were cultured at a constant number of 6 × 10^4^ cells/well either alone (1:0) or at a 4:1 ratio with either 0 Gy or 10 Gy treated iT_REG_ cells 48 h post radiation in a U-bottom 96-well plate with 5 μg/mL plate-bound anti-CD3 and 1 μg/mL anti-CD28 in TexMACS media for 5 days. Proliferation was determined by CFSE dilution on the flow cytometer and analyzed using FlowJo software.

### Statistical analysis

Statistical differences between groups were calculated using the Student *t* test or a one-way ANOVA with Tukey’s post-hoc analysis for multiple comparisons using GraphPad Prism software. Statistical significance was defined as *P* ≤ 0.05. *P* values: *, *P* ≤ 0.05; **, *P* ≤ 0.01; ***, *P* ≤ 0.001.

## Data Availability

The datasets used and/or analysed during the current study are available from the corresponding author on reasonable request.

## Abbreviations

RT: Radiotherapy
T_REG_: Regulatory T cell
T_CONV_: T conventional cell
PBMCs: Peripheral blood mononuclear cells
TGF-β1: Transforming growth factor beta 1
ATRA: All-trans retinoic acid
7-AAD: 7-Aminoactinomycin D
CFSE: Carboxyfluorescein succinimidyl ester
CTLA-4: Cytotoxic T lymphocyte associated protein 4
LAG-3: Lymphocyte activation gene 3
PD-L1: Programmed death ligand 1
MFI: Median fluorescence intensity
CNS2: Conserved noncoding sequence 2
GITR: Glucocorticoid- induced tumor necrosis factor receptor
Egr2: Early growth response gene 2
ICB: Immune checkpoint blockade
