# Correction to: Ionizing radiation modulates the phenotype and function of human CD4+ induced regulatory T cells

**DOI:** 10.1186/s12865-020-00363-y

**Published:** 2020-06-15

**Authors:** Samantha S. Beauford, Anita Kumari, Charlie Garnett-Benson

**Affiliations:** Department of Biology, Georgia State University, 161 Jesse Hill Jr. Dr, Atlanta, GA 30303 USA

**Correction to: BMC Immunol 21, 18 (2020)**

**https://doi.org/10.1186/s12865-020-00349-w**

It was highlighted that in the original article [1] some of the bar graphs of Fig. 4 were blank. This Correction article shows the correct Fig. 4. The original article has been updated. The Publisher would like to apologize to the authors and readers for the inconvenience.

**Fig. 4:**
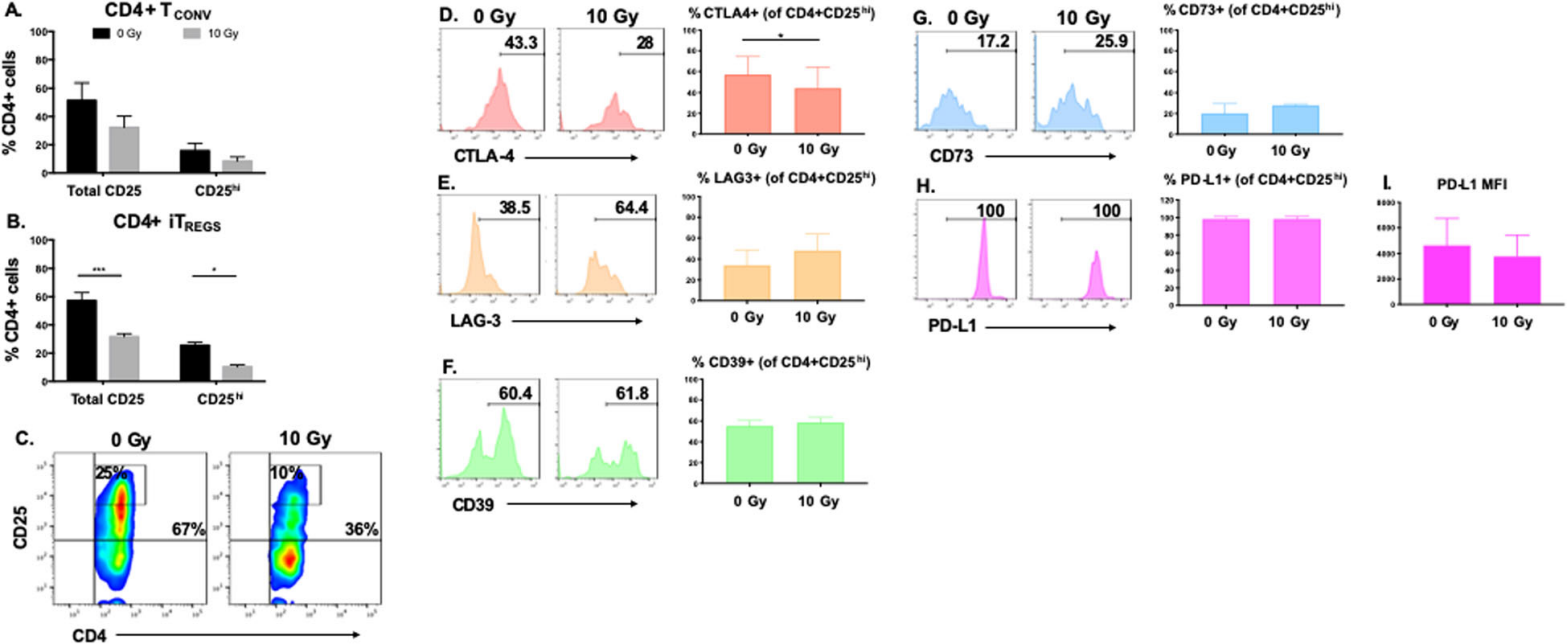
Phenotypic modulation of CD4 + CD25^hi^ iT_REGS_ by radiation. **a** CD4+ T_CONV_ cells or **b** iT_REG_ cells were mock irradiated or exposed to 10 Gy of radiation. Forty-eight hours post treatment CD4+ cells were analyzed for expression of CD25 by flow cytometry. **c** Representative plots of CD4 + CD25+ total (quadrant) or CD4 + CD25^hi^ cells (box with numbers inset in plot). The expression of (**d**) CTLA-4, (**e**) LAG-3, (**f**) CD39, (**g**) CD73, (**h**) PD-L1, and (**i**) PD-L1 mean fluorescence intensity (MFI) were evaluated within the CD4 + CD25^hi^ population 48 h after radiation*. Experiment was repeated three times with similar results. Error bars represent SEM.* *P ≤ 0.05; ** *P* ≤ 0.01; *** *P* ≤ 0.001 by paired, two-tailed Student *t* test

## References

[1] Beauford (2020). Ionizing radiation modulates the phenotype and function of human CD4+ induced regulatory T cells. BMC Immunol.

